# Psychosocial risk factors for impaired health-related quality of life in living kidney donors: results from the ELIPSY prospective study

**DOI:** 10.1038/s41598-020-78032-8

**Published:** 2020-12-07

**Authors:** Ana Menjivar, Xavier Torres, Marti Manyalich, Ingela Fehrman-Ekholm, Christina Papachristou, Erika de Sousa-Amorim, David Paredes, Christian Hiesse, Levent Yucetin, Federico Oppenheimer, Entela Kondi, Josep Maria Peri, Niclas Kvarnström, Chloë Ballesté, Leonidio Dias, Inês C. Frade, Alice Lopes, Fritz Diekmann, Ignacio Revuelta

**Affiliations:** 1 Medical School, University of Barcelona, Barcelona, Spain; 2 Laboratori Experimental de Nefrologia I Trasplantament (LENIT), Institut d’Investigacions Biomediques August Pi i Sunyer (IDIBAPS), Barcelona, Spain; 3 Psychiatry and Clinical Psychology Service, Institut Clinic de Neurociencies, Hospital Clinic of Barcelona, Barcelona, Spain; 4 Transplant Assessorial Unit, Medical Direction, Hospital Clinic of Barcelona, Barcelona, Spain; 5 Karolinska Institutet, Transplantation Surgery, Karolinska University Hospital, Stockholm, Sweden; 6 Department for Internal Medicine and Psychosomatics, Charité, University Medicine, Berlin, Germany; 7 School of Psychology, Aristotle University of Thessaloniki, Thessaloníki, Greece; 8 Department of Nephrology and Renal Transplant, Hospital Clinic of Barcelona, Barcelona, Spain; 9 Donation and Transplant Coordination Section, Hospital Clinic of Barcelona, Barcelona, Spain; 10 Service de Néphrologie et de Transplantation Rénale, Hôpital Foch, Suresnes Cedex, France; 11 Organ Transplant Coordination, Antalya Medical Park Hospital, Antalya, Turkey; 12 Department of Transplantation, Sahlgrenska Academy, University of Gothenburg, Gothenburg, Sweden; 13 Nephrology and Transplant Departments, Hospital Geral de Santo António, Porto, Portugal; 14 Liaison-Psychiatry and Health Psychology Unit, Hospital Geral de Santo António, Porto, Portugal

**Keywords:** Psychology, Medical research, Nephrology

## Abstract

Living kidney donors’ follow-up is usually focused on the assessment of the surgical and medical outcomes. Whilst the psychosocial follow-up is advocated in literature. It is still not entirely clear which exact psychosocial factors are related to a poor psychosocial outcome of donors. The aim of our study is to prospectively assess the donors’ psychosocial risks factors to impaired health-related quality of life at 1-year post-donation and link their psychosocial profile before donation with their respective outcomes. The influence of the recipient’s medical outcomes on their donor’s psychosocial outcome was also examined. Sixty donors completed a battery of standardized psychometric instruments (quality of life, mental health, coping strategies, personality, socio-economic status), and ad hoc items regarding the donation process (e.g., motivations for donation, decision-making, risk assessment, and donor-recipient relationship). Donors’ 1-year psychosocial follow-up was favorable and comparable with the general population. So far, cluster-analysis identified a subgroup of donors (28%) with a post-donation reduction of their health-related quality of life. This subgroup expressed comparatively to the rest, the need for more pre-donation information regarding surgery risks, and elevated fear of losing the recipient and commitment to stop their suffering.

## Introduction

Living kidney donor transplantation (LKDT) represents around the 40% of the overall transplantation activity^1,2^ and is the best treatment currently available for patients with end-stage renal disease. However, the recipient’s benefits must be balanced against potential negative short and long-term physical and psychosocial consequences for living kidney donors (LKDs)^3,4^.

LKDs follow-up is usually focused on the assessment of surgical and medical outcomes, including physical risks. Whilst the psychosocial follow-up is advocated in literature, it remains poorly standardized, and mainly based on cross-sectional studies. Hence, LKDs post-donation psychosocial outcome is still an open topic^5–8^. A more detailed understanding of the potential psychosocial benefits and harms of LKDT is critical to improve the informed consent process and to guarantee the security of the donation process^3,9^.

Available studies on LKDs psychosocial outcomes are commonly retrospective, include single centers with small sample sizes, and are usually based on professional opinions and experiences instead of standardized questionnaires. Therefore, their conclusions might be limited^10^. In spite of recent publications, prospective studies about psychosocial outcomes of donation remains poorly understood^9,11–15^. The results obtained so far suggest that the majority of donors recall the donation experience as a worthy undertaking^12^. However, a small proportion of LKDs report adverse psychosocial and emotional outcomes, mainly depression, anxiety, fatigue, marital stress, and economic costs^3,15–17^. No matter how small the group of LKDs suffering adverse psychosocial outcomes might be, these findings show some room for improvement in current practices. For instance, there is a wide heterogeneity among the transplant centers and a lack of consensus concerning methodology, professionals, instruments, and the most appropriate time to perform the psychosocial pre-donation evaluation and follow-up^8,18–22^. Therefore, both the Transplant Community and International Societies recognize either the importance of replicating the results of prospective and retrospective studies, or of promoting additional ones.

A more precise definition of these risk factors might guide the implementation of interventions to prevent negative consequences and thus the improvement of the information provided to potential donors during the pre-donation assessment^12,21–25^.

This background prompted the design of the European Living Donor Psychosocial Follow-Up (ELIPSY) project, which was a multi-center international research co-funded by the Executive Agency for Health and Consumers grant agreement 20081104. The ELIPSY project aimed to examine the psychosocial outcome and the impact of the donation process on LKDs ensuring a high quality of LKDT programs^19^. One of the ELIPSY project’s branches aimed to prospectively assess the LKDs health-related quality of life (HRQoL) outcome at 1-year post-donation; with the goal of linking their psychosocial profile prior to donation (quality of life, mental health, coping strategies, personality, socio-economic status, psychiatric history, motivations for donation, decision making, and risk assessment) with their respective psychosocial outcome (quality of life, mental health, coping strategies, socio-economic status, life events, donor-recipient relationship, and perception of the recipient’s health). The influence of the recipient's outcome on their LKDs HRQoL was also included in the study.

According to previous authors, we expected that LKDs with a post-donation reduction of their HRQoL would be those who had an impaired post-donation physical health^11,15,26^, higher depression and anxiety levels^11,27^ and lowest dispositional optimism^28^. Regarding recipients’ characteristics, we hypothesize that those LKD´s with recipients with poor medical outcomes will report a higher incidence of HRQoL reduction^11,29^.

## Results

### Participants

**Figure 1:**
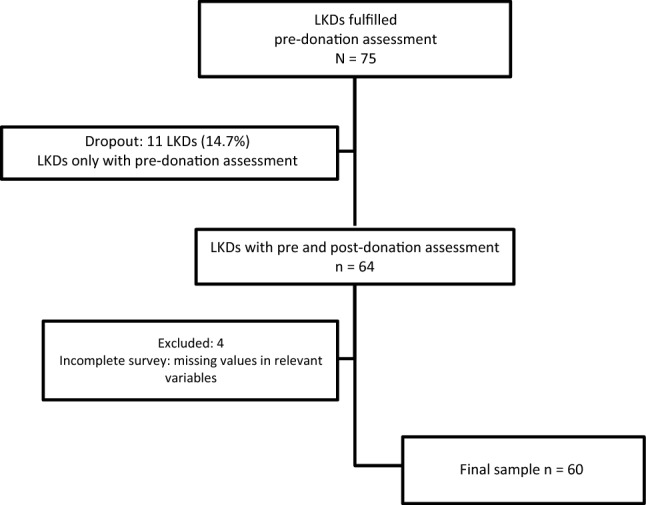
Participants flowchart.

Seventy-five LKDs were included at pre-donation. All donors were genetically or emotionally related with their recipient. The range of LKDs participation was comparable between centers, the percentages varied from 26.3 to 34.9%. Sixty-four (85.3%) LKDs completed the post-donation follow-up while 11 (14.7%) were lost during the follow-up. At post-donation, four LKDs out of the 64 were excluded, due to missing values in relevant variables. The final sample was 60 LKDs, and they will be referred to as completers (Fig. 1).

### Principal component analysis of motivations for donation

Table 1 shows that 26 out of 31 items assessed by Likert scale were included in the analysis. Every item produced a range of responses from 1 (not relevant) to 5 (very important).

**Table 1:** Principal component analysis of motivations for donation.

Items^†^	Factor			
	1	2	3	4
114—Fear that giving up my kidney might shorten my life span	0.030	0.244	**0.663**	0.227
116—Fear that giving up my kidney might damage my health or cause me problems in the future	− 0.043	0.201	**0.769**	0.070
117—Concern that some of my family or friends disapprove of my donating my kidney	0.223	− 0.262	**0.786**	0.186
118—Concern that some of my family or friends would disapprove of my decision not to donate	0.291	− 0.251	**0.717**	0.227
119—Wish to feel closer to the person that I was donating my kidney	0.090	0.191	0.166	**0.663**
121—Concern about having a scar	− 0.140	0.148	**0.605**	− 0.129
122—Concern about financial implications of donating my kidney	− 0.243	− 0.224	0.223	**0.553**
123—Feeling that this was a unique opportunity to do something very special	0.161	0.127	− 0.093	**0.727**
124—Wish to feel that I am a good person	**0.430**	− 0.008	0.061	**0.721**
125—Worry that the person I was donating my kidney to might not take care of it properly	**0.479**	0.155	**0.639**	− 0.044
126—I didn’t want to lose the recipient, I am afraid he/she will die if I do not donate	0.025	**0.724**	0.063	0.039
128—I am unable to watch the recipient suffer and not do anything about it	0.067	**0.570**	− 0.024	− 0.025
129—It is a personal challenge/test for me	0.212	0.279	0.130	**0.635**
130—Wish to show my deep love and respect to the recipient	0.021	**0.517**	0.073	**0.557**
131—Feeling responsible for the recipient	**0.400**	**0.508**	0.074	0.244
132—Feeling I owe that to the recipient	**0.755**	0.173	0.027	0.184
133—Wish not to disappoint the recipient	**0.680**	0.135	0.036	0.117
135—In past situations, I did not have the chance to influence things. Now I do and I want to use this chance	− 0.100	**0.464**	0.239	0.091
136—The recipient is too young to suffer/die and deserves to live further	0.247	**0.488**	0.099	− 0.281
137—Feeling obligated as a person/relative to do that	**0.827**	0.114	− 0.071	0.030
138—Feeling of being responsible as a family member to do that	**0.793**	0.031	0.063	0.097
139—I am the only donor available	0.337	**0.646**	0.016	0.234
140—I am the most suitable donor	0.330	**0.471**	0.093	0.168
141—There is no other choice	**0.452**	**0.630**	− 0.058	0.045
142—The family thought/decided that I would be the most suitable to donate	**0.590**	0.087	0.252	0.029
143—It just makes sense for me to donate	0.004	**0.551**	0.005	0.162

Bold values are the items cross-loaded in each factor.

^†^Likert scale for all the items from 1 (not relevant) to 5 (very important).

The main component analysis with varimax rotation led to the extraction of four-factors, accounting for 53.7% of the variance in the item-set. Factors accounted for 15.6%, 14.2%, 12.4%, and 11.5% of variance respectively. Five items (#113, #115, #120, #127 and #134) did not contribute to the factors definition and thus were excluded.

Factor 1 included nine items assessing LKDs’ personal responsibility, and obligation. Factor 2 included 10 items describing the donors’ fear of losing the recipient/commitment to stop their suffering/save their lives. Factor 3 included six items measuring LKDs’ fears and concerns about donations’ own consequences and family and friends’ opinion; including the lifelong consequences of donating a kidney. Factor 4 included six items assessing the expectations/goals to fulfill with the donation decision; covering personal growth, interpersonal benefits, and the financial implications of donation. Five items (#124, #125, #130, #131 and #141) showed positive high-loads in two factors. Therefore, these items were included in both factors.

Internal consistency was: Alpha for Factor 1 = 0.84; Factor 2 = 0.82; Factor 3 = 0.79; Factor 4 = 0.78.

### Randomness of attrition

Differences between completers (60) and LKDs lost to follow-up (11) for donor and recipient characteristics were analyzed. Completers showed lower levels of depression and higher scores in the EPQ-RA extraversion scale. Concerning the recipients’ characteristics, Diabetes Mellitus as indication for kidney transplantation was significantly less frequent in the completers group.

### Pre-donation characteristics

Table 2 describes the completers and their recipients’ baseline characteristics. Most completers were women (60.0%), with a mean (SD) age of 50.2 (11.7) years at donation, the majority had a partner (63.3%), and were genetically related with their recipient (53.3%).

**Table 2:** Pre-donation characteristics and differences between clusters.

	All partici-pants (n = 60)	Cluster 1 (n = 43)	Cluster 2 (n = 17)	t/x^2^	*p*	Univariate logistic regression* p*^§^
	Mean (sd)/n (%)	Mean (sd)/n (%)	Mean (sd)/n (%)			
**Socio-demographic characteristics**						
Age (years)	50.18 (11.73)	51.41 (11.40)	47.05 (12.31)	1.306	0.197	**0.191**
Gender (female)	36 (60.0%)	25 (58.1%)	11 (64.7%)	0.219	0.640	0.638
Donor-recipient relationship (genetically related)	32 (53.3%)	24 (55.8%)	8 (47.1%)	0.375	0.540	0.541
**Marital status**						
Without partner (single, divorced, widowed)	22 (36.7%)	14 (32.6%)	8 (47.1%)	1.140	0.565	**0.298**
With partner	38 (63.3%)	29 (67.4%)	9 (52.9%)			
Level of studies						
No academic, elementary	19 (32.2%)	13 (31.0%)	6 (35.3%)	0.104	0.747	0.748
Intermediate	22 (37.3%)	15 (35.7%)	7 (41.2%)	0.154	0.694	0.695
Upper studies	18 (30.5%)	14 (33.3%)	4 (23.5%)	0.549	0.545^†^	0.452
**Psychometric instruments**						
Quality of life						
Anamnestic comparative self-assessment, range − 5 (worst period) to 5 (best period)	2.79 (1.74)	2.59 (1.85)	3.29 (1.33)	− 1.335	0.188	**0.158**
**Item short form survey (SF-36), range 0–100**^¶^						
Physical functioning (PF)	53.87 (5.45)	55.09 (3.35)	50.78 (8.12)	2.119^‡^	**0.048**	
Role physical (RP)	55.09 (3.01)	55.02 (3.29)	55.26 (2.21)	− 0.283	0.778	
Bodily pain (BP)	58.43 (5.28)	58.69 (4.59)	57.78 (6.85)	0.504^‡^	0.619	
General health (GH)	56.28 (7.01)	57.05 (6.34)	54.32 (8.36)	1.216^‡^	0.236	
Vitality (VT)	58.00 (7.47)	57.40 (7.81)	59.54 (6.51)	− 0.999	0.322	
Social functioning (SF)	54.90 (4.83)	54.72(5.45)	55.36 (2.77)	− 0.459	0.648	
Role emotional (RE)	54.60 (3.64)	54.37 (4.24)	55.17 (1.04)	− 0.762	0.449	
Mental health (MH)	55.48 (6.58)	54.40 (6.94)	58.21 (4.71)	− 2.078	**0.042**	
**Mental health status**						
Hospital anxiety and depression scale						
Anxiety scale, range 0–21	3.70 (2.84)	3.91 (2.88)	3.18 (2.74)	0.892	0.376	0.358
Depression scale, range 0–21	1.75 (1.94)	1.93 (2.04)	1.29 (1.65)	1.146	0.257	**0.231**
**Coping strategies**						
Optimism, life orientation test-revised (sum of items) range 6–30	23.90 (4.44)	24.14 (4.58)	23.29 (4.17)	0.661	0.511	0.508
Sense of coherence, range 1–7	5.55 (0.94)	5.56 (0.90)	5.52 (1.07)	0.146	0.885	0.882
**Personality**						
Eysenck personality questionnaire-revised-abbreviated						
Neuroticism scale, range 0–6	0.98 (1.20)	1.00 (1.25)	0.94 (1.06)	0.177	0.860	0.857
Extraversion scale, range 0–6	4.10 (1.76)	4.14 (1.78)	4.00 (1.79)	0.273	0.786	0.782
Psychoticism scale, range 0–6	1.41 (1.07)	1.33 (1.11)	1.63 (0.96)	− 0.956	0.343	0.334
**Socioeconomic status**						
English longitudinal study of ageing range 1 (worst off) to 10 (best off)						
Socioeconomic status range 1 (worst off) to 10 (best off)	6.31 (1.43)	6.35 (1.42)	6.20 (1.47)	0.345	0.732	0.726
**Ad hoc items**						
Psychiatric history						
Have you ever seen a counselor, psychiatrist or psychologist? *(yes)*	11 (18.6%)	10 (23.3%)	1 (6.3%)	2.223	0.259^†^	**0.104**
**Motivations/reasons for donation**						
Personal responsibility and obligation *(mean of items)* range 1 (not relevant) to 5 (very important)	2.07 (0.89)	1.92 (0.77)	2.47 (1.07)	− 2.081	**0.043**	**0.046**
Donors’ desire to stop their recipients’ suffering and/or save their lives *(mean of items)* range 1 (not relevant) to 5 (very important)	3.40 (0.90)	3.27 (0.87)	3.87 (0.83)	− 2.313	**0.025**	**0.022**
Donors’ fears and concerns about donation’s own consequences and family and friends opinion *(mean of items)* range 1 (not relevant) to 5 (very important)	1.33 (0.55)	1.27 (0.46)	1.47 (0.71)	− 1.229	0.224	**0.236**
Challenges involving the donation decision; including personal growth and financial implications *(mean of items)* Range 1 (not relevant) to 5 (very important)	2.42 (0.99)	2.33 (1.03)	2.64 (0.86)	− 1.040	0.303	**0.297**
**Decision making**						
Necessity of some time to think over donation						
Strongly disagree/Disagree	35 (60.3%)	26 (63.4%)	9 (52.9%)	0.551	0.458	0.460
Agree/strongly agree	23 (39.7%)	15 (36.6%)	8 (47.1%)			
**Risk assessment**						
Necessity of more information regarding the surgery and its risks (*yes*)	6 (10.2%)	2 (4.8%)	4 (23.5%)	4.667	0.097	0.041
**Recipients’ characteristics**						
Recipients’ socio-demographic characteristics						
Weight (Kg)	77.64 (19.07)	79.37 (20.33)	73.09 (14.89)	1.122	0.267	**0.256**
**Recipient baseline**						
African ancestry (yes)	2 (3.4%)	1 (2.4%)	1 (5.9%)	0.453	0.497^†^	0.521
Indication of transplantation						
Urological	4 (6.9%)	4 (9.5%)	0 (0%)	1.637	0.567^†^	
Glomerulopathy	17 (29.3%)	14 (33.4%)	3 (18.8%)	1.189	0.347^†^	
Nephroangioesclerosis	7 (12.1%)	4 (9.5%)	3 (18.7%)	0.929	0.381^†^	
Diabetes Mellitus	2 (3.4%)	0 (0%)	2 (12.5%)	5.438	0.073^†^	
Others	8 (13.8%)	6 (14.3%)	2 (12.5%)	0.031	1.000^†^	
Unknown	12 (20.7%)	10 (23.8%)	2 (12.5%)	0.903	0.479^†^	
Polycystic kidney disease	8 (13.8%)	4 (9.5%)	4 (25.0%)	2.334	0.198^†^	
Registration in waiting list for deceased donor (yes)	35 (60.4%)	22 (53.7%)	13 (76.5%)	2.613	0.144^†^	**0.098**
Dialysis before transplantation (yes)	39 (67.2%)	27 (65.9%)	12 (70.6%)	0.122	0.727	0.725
Previous kidney transplantation (yes)	6 (10.3%)	5 (12.2%)	1 (5.9%)	0.516	0.660^†^	0.451
Previous other organ transplantation (yes)	0 (0%)	0 (0%)	0 (0%)			
High immunological risk (yes)	11 (19.6%)	7 (17.1%)	4 (26.7%)	0.640	0.461^†^	0.434
High risk of recurrence (yes)	4 (7.0%)	3 (7.3%)	1 (6.3%)	0.020	1.000^†^	0.886
Presence of serious co-morbid diseases (yes)	11 (19.3%)	8 (19.5%)	3 (18.8%)	0.004	1.000^†^	0.948

^†^Fisher’s exact test.

^‡^Not assuming equality of variances.

^§^Any p-value remained statistically significant after FDR correction.

^¶^SF-36 T scores (> 55 is higher than average, < 45 is lower than average).

LKDs’ baseline of SF-36 T-scores was in the average of the general population on all eight dimensions (scale 0–100). Only 11 donors (18.6%) had seen a psychiatrist or psychologist before.

In relation to the decision-making process, most of the LKDs (60.3%) did not consider it was necessary to take time to think over the donation and concerns to risk assessment, and the majority (89.8%) did not need more information regarding the surgery and its risks.

The recipients’ most frequent indication for transplantation was glomerulopathy (29.3%); the majority was registered in a waiting list for deceased donor (60.4%) and was on dialysis before transplantation (67.2%).

### Post-donation outcomes

Table 3 shows the post-donation outcomes of LKDs and their recipients. At post-donation, mental health remained stable for the majority of donors. Few adverse psychosocial outcomes were observed; regarding their employment situation, only one donor (1.8%) reported being unemployed because of donation. The donor-recipient relationship had deteriorated in one case (1.7%) and only one donor (1.7%) required psychological/psychiatric treatment after donation. However, nearly a quarter (22.4%) of the LKDs self-reported physical complications related with the donation (e.g. scars, pain, etc.).

**Table 3:** Post-donation characteristics and differences between clusters.

	All partici-pants (n = 60)	Cluster 1 (n = 43)	Cluster 2 (n = 17)	t/x^2^	*p*
	Mean (SD)/n (%)	Mean (SD)/n (%)	Mean (SD)/n (%)		
Elapsed time between donation and donors’ follow-up (months)	12.78 (3.44)	13.34 (3.44)	11.36 (3.12)	2.069	**0.043**
**Psychometric instruments**					
**Quality of life**					
Anamnestic comparative self-assessment, range − 5 (worst period) to 5 (best period)	2.77 (1.79)	3.07 (1,68)	1.98 (1.88)	2.157	**0.035**
36-item short form survey (SF-36) range 0–100^§^					
Physical functioning (PF)	52.79 (7.32)	55.54 (3.04)	45.84 (10.10)	3.890^‡^	**0.001**
Role Physical (RP)	53.13 (7.31)	55.37 (2.24)	47.45 (11.67)	2.779^‡^	**0.013**
Bodily pain (BP)	54.46 (8.76)	58.84 (4.00)	43.36 (7.66)	7.915^‡^	** < 0.001**
General health (GH)	54.71 (8.53)	57.50 (7.22)	47.64 (7.61)	4.695	** < 0.001**
Vitality (VT)	54.51 (7.89)	56.31 (7.24)	49.98 (7.82)	2.985	**0.004**
Social functioning (SF)	54.37 (4.70)	55.35 (3.72)	51.88 (6.01)	2.215^‡^	**0.038**
Role emotional (RE)	52.63 (7.32)	54.18 (4.47)	48.70 (11.05)	1.982^‡^	0.063
Mental health (MH)	54.30 (6.48)	55.90 (4.71)	50.28 (8.54)	2.563^‡^	**0.019**
**Mental health status**					
Hospital anxiety and depression scale					
Anxiety scale, range 0–21	3.73 (3.18)	3.11 (2.97)	5.24 (3.25)	− 2.412	**0.019**
Depression scale, range 0–21	1.17 (1.75)	0.85 (1.33)	1.94 (2.36)	− 1.787^‡^	0.089
**Coping strategies**					
Sense of coherence, range 1–7	5.63 (0.84)	5.72 (0.83)	5.40 (0.83)	1.366	0.177
**Social support**					
English longitudinal study of ageing					
Socioeconomic status range 1 (worst off) to 10 (best off)	6.64 (1.54)	6.70 (1.46)	6.47 (1.81)	0.497	0.621
**Ad hoc items**					
**Quality of life**					
Suffered complaints or illnesses because of the donation *(Yes)*	13 (22.4%)	3 (7.3%)	10 (58.8%)	18.333	** < 0.001**
Current physical condition affected by the donation. range 0 (nothing) to 10 (a lot)	1.84 (2.67)	1.44 (2.63)	2.88 (2.58)	− 1.864	0.068
The post-operative recovery was:					
More difficult than I had imagined	14 (24.1%)	7 (17.1%)	7 (41.2%)	3.813	0.051
Easier than I had imagined	29 (50.0%)	20 (48.8%)	9 (52.9%)	0.083	0.773
Exactly as I had imagined	14 (24.1%)	13 (31.7%)	1 (5.9%)	4.377	**0.046**^**†**^
I don’t know/I don’t remember	1 (1.8%)	1 (2.4%)	0 (0%)	0.422	1.000^†^
**Mental health**					
Necessity of psychological/psychiatric treatment or counseling since the donation					
No	57 (95.0%)	40 (93.0%)	17 (100%)	1.248	0.551^†^
Yes	2 (3.3%)	2(4.7%)	0 (0%)	0.818	1.000^†^
Yes, because of the donation	1 (1.7%)	1 (2.3%)	0 (0%)	0.401	1.000^†^
Current emotional condition affected by the donation. range 0 (nothing) to 10 (a lot)	1.75 (2.84)	1.40 (2.69)	2.65 (3.01)	− 1.555	0.126
After surgery I felt forgotten *(true)*	7 (11.7%)	3 (7.0%)	4 (23.5%)	3.239	0.092^†^
Somehow I feel worried about my health since the donation *(true)*	5 (8.5%)	1 (2.4%)	4 (25.5%)	6.978	**0.021**^**†**^
The recipient’s health still occupies me a lot *(true)*	46 (78.0%)	32 (76.2%)	14 (82.4%)	0.268	0.738^†^
**Socio-economic status**					
Did you suffer any financial loss due to the donation? *(Yes)*	20 (33.3%)	15 (34.9%)	5 (29.4%)	0.164	0.685
**Life events**					
Did you experience any other life changing events since donation? *(yes)*	13 (22.0%)	10 (23.3%)	3 (18.8%)	0.138	1.000^†^
Intensity of distress for life events *(Mean of items)* Range 0 (no distress at all) to 10 (the worst experience)	4.23 (5.3)	4.12 (5.5)	4.7 (4.6)	− 0.265	0.792
**Donor-recipient relationship**					
Do you feel your relationship to the recipient has changed after the donation? *(yes)*	19 (31.7%)	14 (32.6%)	5 (29.4%)	0.056	0.813
Have you been in charge of the recipient since the donation? *(yes)*	18 (31.6%)	13 (30.2%)	5 (35.7%)	0.147	0.702
**Perception of the recipient’s health after transplantation**					
The recipient enjoys good health currently					
Strongly disagree/disagree	5 (8.5%)	2 (4.7%)	3 (18.8%)	2.988	0.118^†^
Agree/strongly agree	54 (91.5%)	45 (95.3%)	13 (81.2%)		
The recipient of my organ behaves in a way that risks the continued healthy functioning of the donated kidney					
Strongly disagree/disagree	54 (93.1%)	38 (90.5%)	16 (100%)	1.637	0.567^†^
Agree/strongly agree	4 (6.9%)	4 (9.5%)	0 (0%)		
**Recipients’ post-transplant complications**					
Permanent damage in organ function (yes)	2 (3.5%)	0 (0%)	2 (11.7%)	4.996	0.082^†^
Inpatient hospitalization or Prolongation of hospitalization (yes)	21 (36.2%)	14 (33.3%)	7 (43.8%)	0.544	0.461
Permanent or temporary discapacity not requiring hospitalization (yes)	7 (13.0%)	5 (11.9%)	2 (16.7%)	0.188	0.645^†^
Treatment non-adherence (yes) n:48	1 (2.1%)	1 (2.8%)	0 (0%)	0.340	1.000^†^
Psychological complications requiring treatment (yes) n:39	1 (2.6%)	0 (0%)	1 (12.5%)	3.977	0.205^†^
Serum creatinine (Units)	131.7 (98.3)	122.4 (42.0)	157.7 (180.2)	− 0.753^‡^	0.464
Recipient survival (yes)	58 (98.3%)	41 (97.6%)	17 (100%)	0.412	1.000^†^
Graft survival (yes)	56 (94.9%)	41 (97.6%)	15 (88.2%)	2.208	0.197^†^

^†^Fisher’s exact test.

^‡^Not assuming equality of variances.

^§^SF-36 T scores (> 55 is higher than average, < 45 is lower than average).

The recipient and graft survival at 1-year was 98.3% and 94.9% respectively. Two (3.5%) recipients had unexpectedly bad kidney function (glomerular filtration rate below 30 mL/min) at 1-year. The reported serum creatinine was within the expected ranges (131.7 (98.3) µmol/L).

### Comparison between pre and post-donation

Table 4 shows the comparative results between pre and post-donation assessments. SF-36 T-scores worsened at post-donation on: limitations due to physical problems (role physical), pain and the effect of pain on activities (bodily pain), and the feeling of energy (vitality). HADS depression dimension showed a statistically significant decrement at post-donation. ELSA subjective socioeconomic status reported a small, but statistically significant, post-donation improvement.

**Table 4:** Differences between pre-donation and post-donation assessment.

	Pre-donation (n = 60)	Post-donation (n = 60)	Post vs. pre-donation		
	Mean (SD)	Mean (SD)	Difference	t	*p*
**Quality of life**					
ACSA range − 5 (worst period) to 5 (best period) n:51	2.75 (1.73)	2.74 (1.84)	0.01 (2.27)	0.049	0.961
36-item short form survey (SF-36), range 0–100^†^					
Physical functioning (PF)	53.87 (5.45)	52.79 (7.32)	1.08 (6.94)	1.208	0.232
Role physical (RP)	55.09 (3.01)	53.13 (7.31)	1.96 (7.29)	2.084	**0.041**
Bodily pain (BP)	58.43 (5.28)	54.46 (8.76)	3.97 (8.64)	3.560	**0.001**
General health (GH)	56.28 (7.01)	54.71 (8.53)	1.57 (8.73)	1.395	0.168
Vitality (VT)	58.00 (7.47)	54.51 (7.89)	3.49 (7.77)	3.479	**0.001**
Social functioning (SF)	54.90 (4.83)	54.37 (4.70)	0.53 (6.63)	0.621	0.537
Role emotional (RE)	54.60 (3.64)	52.63 (7.32)	1.97 (7.91)	1.926	0.059
Mental health (MH)	55.48 (6.58)	54.30 (6.48)	1.18 (7.89)	1.155	0.253
**Mental health status**					
Hospital anxiety and depression scale					
Anxiety scale	3.68 (2.78)	3.80 (3.17)	− 0.11 (3.17)	− 0.272	0.787
Depression scale	1.76 (1.96)	1.19 (1.76)	0.56(2.05)	2.076	**0.042**
**Coping strategies**					
Sense of coherence, range 1–7	5.55 (0.94)	5.63 (0.84)	− 0.08 (0.61)	− 1.026	0.309
**Social support**					
English longitudinal study of ageing					
Socioeconomic status, range 1 (worst off) to 10 (best off) n:53	6.30 (1.42)	6.77 (1.50)	− 0.47 (1.38)	− 2.486	**0.016**

^†^SF-36 T scores (> 55 is higher than average, < 45 is lower than average).

### Cluster-analyses

**Figure 2:**
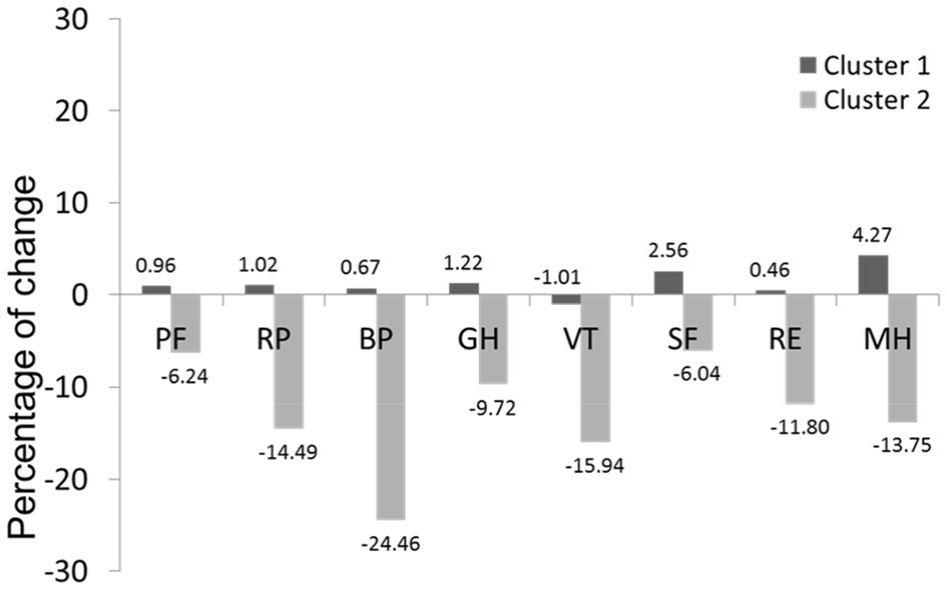
Percentage of change in the SF-36 by clusters. *PF* physical functioning, *RP* role physical, *BP* body pain, *GH* general health, *VT* vitality, *SF* social functioning, *RE* role emotional, *MH* mental health.

The two-step cluster-analysis suggested two subgroups of LKDs: cluster 1 (n = 43, 71.7%) reported no change in their HRQoL, and cluster 2 (n = 17, 28.3%) reported a post-donation worsening of their HRQoL. Percentages of change between clusters are shown in Fig. 2. Donors from cluster 2 were characterized by a statistically significant decrement on five of the eight scales of SF-36: limitations due to physical problems (role physical), pain and the effect of pain on daily activities (bodily pain), the energy (vitality), limitations due to emotional problems (role emotional), and psychological distress and well-being (general mental health).

### Cross-sectional comparisons between clusters

At pre-donation, donors from cluster 1 and 2 were similar in most socio-demographic and clinical characteristics. However, cluster 2 was characterized by a higher motivation to donate because of feeling responsible as recipient’s relative and/or feeling that there was no other choice, because of fear of losing the recipient and emotional commitment to stop their suffering/safe their lives, and a higher necessity of more information regarding the surgery and its risks (Table 2).

At post-donation, cluster 2 was characterized by: shorter elapsed time from donation to post-assessment, a lower QoL using ACSA, and higher scores on HADS anxiety dimension. In addition, a higher proportion of LKDs in cluster 2 reported to still suffering complaints and/or illnesses due to donation, considered the post-operative recovery more difficult than they expected and a higher proportion of donors’ feel worried about their health since the donation. The two recipients with permanent damage in organ function were related to donors in cluster 2 (Table 3).

### Model of pre-donation assessment

The univariate logistic regressions for each independent variable showed that 12 potential predictive factors reached the level of statistical significance below the 0.30 required as screening criterion. These factors included socio-demographic characteristics (age and marital status), QoL and mental health status (ACSA and HADS depression dimension), psychiatric history, the four motivations for donation, risk assessment (necessity of more information regarding the surgery and its risks), and recipient characteristics (recipients’ weight and waiting list registration for deceased donor). Table 2 shows the results of the univariate logistic regression.

All these potential predictive factors were assessed by multivariate logistic regression. Results showed that the pre-donation profile of the LKDs showing a decrease of their HRQoL after donation (Cluster 2) was characterized by the feeling of needing more information regarding the surgery and its risks, and by donating because of fear of losing the recipient/commitment to stop their suffering/safe their lives (Table 5). The sample size was reduced to 59 donors, due to one participant had a missing value on the measure “Fear of losing the recipient/commitment to stop their suffering/save lives”. As there was only one LKD, we decided to not make any kind of imputation.

**Table 5:** Logistic regression for the model of pre-donation assessment (n = 59)^†^.

	B	S.E	Wald	*df*	*p*	Exp (B) (95% CI)
Necessity of more information regarding the surgery and its risks	2.157	1.001	4.643	1	0.031	8.467 (1.215 61.532)
Fear of losing the recipient/commitment to stop their suffering/save lives	1.009	0.451	5.009	1	0.025	2.743 (1.134 6.638)

^†^One donor had a missing value on Fear of losing the recipient/commitment to stop their suffering/save lives.

The final pre-donation model with only these two predictors and a modified cut-point of the estimated probability to 0.33 showed a good overall percentage of correct classifications (75.0%), with acceptable sensitivity (69.2%) and specificity (77.1%). The AUC = 79.7% (CI 95% 65.0–89.5%) was good, and the shrinkage was very low when compared with the AUC obtained with bootstrap estimation (AUC_bootstrap_ = 78.5%). The feeling of needing more information (yes/no) regarding the surgery and its risks, multiplied by 8.5 the risk of belonging to cluster 2; compared to those donors who did not need additional information about the surgery process; while every unit increased (range 1–5) the perception of donor’s fear of losing the recipient/commitment to stop their suffering/save their lives as motivation for donation multiplied by 2.7 the risk of presenting a cluster 2 outcome.

## Discussion

As previously observed, our results suggest that taken as a whole, LKDs’ psychosocial status remains stable after donation^3,9,30,31^. Very few post-donation adverse psychosocial outcomes were observed. However, a subgroup of donors, characterized by needing more information regarding surgery risks, and those who donated because the fear of losing the recipient/commitment for stopping their suffering, suffered a slight deterioration of some aspects of their HRQoL. Both variables seem amenable to modification during the donor selection process.

Previous studies have reported that 10–31% of LKDs believe that more information about donation should have been provided pre-operatively^16,32^. The perception of being poorly informed pre-donation has been proposed as a determinant of post-donation donor dissatisfaction, especially when the information received pre-donation and the donors’ actual experiences highly diverge^33^. The perception of needing more information regarding the surgery and its risks should be carefully examined to answer all the potential LKDs’ doubts, and ensure they feel ready for donation. Furthermore, the necessity of more information might be due to inadequate information, but in some cases, it could also be an expression of ambivalence towards donation. This clinical attitude, intended to assure legitimacy of the informed consent process, should also include the disclosure of any risks newly recognized in the literature^15^.

It has already been described that the LKDs’ strongest motive to donate is the desire to improve and/or save their recipients’ life^34^. However, LKDs might not consider some variables related to their recipients’ post-donation behaviors, such as non-adherence to treatment or consuming excessive alcohol, which fall out of their control. These post-donation issues might worsen their experience of donation by causing disappointment or even deterioration of the donor-recipient relationship^35^. Moreover, a donation mainly motivated by the perceived need of saving their recipients’ life may influence LKDs’ decision-making process by speeding up the donation procedure^36^. Similarly, feelings of obligation and being mainly responsible for the recipients’ health can entail aspects of pressure and coercion (e.g. by family) and compromise the autonomy of the donors’ decision and thus their post-operative psychosocial outcome. These scenarios might have caused the donor to feel not ready for donation. Both prompt identification of a prevailing, urgent, and unjustified need to save the recipients’ life, and exploration and resolution of the actual recipients’ vital risk (e.g., by discussing alternative interventions such as dialysis) might help to ameliorate this risk factor. Additional research regarding LKDs’ motivation to donate is needed to replicate our factor solution; we considered that the motivations questionnaire is an interesting tool that could be useful in future studies.

Subjective socioeconomic status reported a post-donation small, but statistically significant, post-donation improvement. This improvement might be related to the amelioration of their recipients’ physical health. For example, two-thirds of our recipients were on dialysis before transplantation. After transplantation, considering that all our donors were genetically or emotionally related, both the recipient and the donor would be released from the out of pocket expenses caused by the dialysis treatment, and the recipient might restart a full working life, which could also have a positive effect on the donors’ socioeconomic status. Although our study did not measure changes in working status, a recent study shows that the percentage of recipients who were back to work 1-year after transplantation depends on the type of work^37^. Additional research is needed both to confirm the improvement in socioeconomic status and to define its determinants.

The comparison between pre and post-donation HRQoL showed a statistical significance on the reduction of three scales of the SF-36. Two of them, measuring the physical component: role physical (the extent to which physical health interferes with work or other daily activities) and bodily pain (intensity of pain and its effect on activities). The last one, vitality, indicates a decline in energy and an increase in fatigue, which has been normally associated to the mental component of HRQoL, but surely, it also affects the physical component. Vitality scores could remain somewhat lower in the long-term, the declination of vitality scores requires further investigation to define if it is a consequence of donation or a combination of multiple factors^30^, and if it is the mostly affected component of HRQoL affected by donation^15^. It is important to highlight, that these reduction was clinically relevant only on bodily pain scale, which was outside of the normal range for the general population.

A higher proportion of donors with HRQoL decrement reported an increased risk of impaired post-donation physical health (still suffering complaints and/or illnesses due to donation and considered the post-operative recovery more difficult than they expected). Donors’ physical complications and the experience with a long recovery time have been associated with impaired function and well-being many years after donation^26^. During the first year post-donation, the LKDs perform a narrower medical follow-up for the prompt identification of medical and psychosocial complications; nonetheless, LKDs with HRQoL decrement feel worried about their health, in a higher percentage since the donation.

Contrary to our expectations, no association between recipients’ medical characteristics (pre and post-transplantation) and donors’ psychosocial outcomes were found. Previous studies have shown that recipients’ outcomes may influence donors’ QoL and mental health. However, their results are contradictory, showing either an adverse influence^11,29,36^, or no association between medical complications of recipients and donors’ psychosocial outcomes^26,31,38^. A closer monitoring of donors with high-risk recipients at pre-donation (e.g. hyperimmunized patients) or with perioperative complications (e.g. bleedings, hematomas, and wound infections) or with long-term complications (e.g. graft failure and recipient’s death) might add important insights to this area. Our recipients’ follow-up is limited to 1-year, and the incidence of graft failure and recipients’ death was low accordingly. Longer recipients’ and donors’ follow-ups are needed to examine the real impact of these variables on LKDs’ psychosocial outcomes^29^.

### Limitations

Data about the surgical techniques (open vs. laparoscopic nephrectomy) was not included in the statistical analysis and we may not be sure whether the inclusion of these variables would change the clusters’ structure. However, available literature suggests that differences in surgical techniques seem poorly related to HRQoL outcomes^39–42^.

We cannot determine whether donors who completed only the pre-donation questionnaire (13.9%), avoided participating in the follow-up process due to the malfunctioning of psychosocial or physical nature. In our study, the completers were characterized by lower levels of depression and higher scores in personality extraversion scale. Depression has been associated with poor adherence to self-care regimens and increased medical complications in patients with chronic medical illness^43^. Also, adaptive personality characteristics such as high extroversion (the tendency to positive affect and interpersonal connection) has been found as the only personality characteristic significantly correlated with post-donation growth^44^. Therefore, we may not discard a “self-selection” bias in our sample with donors with more resilient traits, being more likely to respond the post-donation questionnaire. For this reason, these results need to be considered with some caution.

The follow-up sample size did not allow a segmented analysis by transplant center. Center-specific practices might compromise the generalization of our results (e.g. center policies to select donors with comorbidities and/or countries regulations to limit donation by type of donor-recipient relationship). The inclusion of other centers might increase the number of participants to help solve this issue. Moreover, the limited sample size lowered the statistical power, especially in multivariate analysis.

We used as reference data for SF-36 T-scores the general population, which includes subjects with chronic diseases. As was suggested by previous authors, a comparison with a healthy population might be preferable^11^. However, even a selected healthy cohort might not be adequate, because some LKDs may suffer from hypertension or metabolic disorders before the donation and a healthy population will not undergo a surgical intervention comparable to kidney donation^45^.

The absence of the inclusion of a control group to clarify if the observed changes in LKDs psychosocial sphere also have occurred had they not donated, and if these changes do not differ from the changes observed in the general population as normal fluctuations^13^.

Our cluster 2 was characterized by shorter elapsed time from donation to post-assessment. This difference might be caused by the variability of each participant to answer the post-donation questionnaire (e.g. elapsed time for postal-mail reception or elapsed time between reception and donor completion). It is well recognized that LKDs HRQoL usually returns to baseline over time or remains slightly reduced^3, 30^.

Kidney recipient variables were assessed only in medical terms, socio-demographic and psychosocial variables were not collected. Thus, it is not possible to determinate if the recipients’ psychosocial status (pre and post-transplantation) influenced their donors’ psychosocial outcome.

## Conclusion

LKDs’ do well at 1-year follow-up and their HRQoL is comparable with the general population. However, after donation, a subgroup of donors characterized by feeling somewhat unsure about the surgery and their risks before donation and feeling emotionally involved and committed to ameliorating the suffering of their recipients, showed a worsening of their HRQoL, mainly due to the physical domain.

## Methods

The study was approved by the Ethics Committee of the Hospital Clinic of Barcelona and by the Institutional Ethical Committees at each participant center. The investigation was performed according to the Declaration of Helsinki 2000. All donors provided written informed consent to participate.

### Study design

We conducted a prospective, multicenter longitudinal study. The participating centers were: Hôpital Necker Enfants Malades (France), Charité Universitätsmedizin Berlin (Germany), Centro Hospitalar do Porto (Portugal), Hospital Clinic of Barcelona (Spain), and Sahlgrenska University Hospital (Sweden). The Turkish Medical Park Hospital Antalya contributed on the bibliography revision, and sharing their experiences as one of the largest transplant centers in Europe, performing up to 500 LKDTs per year^46^.

### Participants

LKDs who donated in the participating centers during the timeframe of the study were invited to participate; the inclusion period was 12 months (year 2011). LKDs were excluded if: (a) it was not possible to send back the questionnaire by postal-mail because the donor did not live in the transplant center’s country, (b) were illiterate, (c) did not understand the transplant country’s main language.

### Instruments

The research team identified and get consensus about the most relevant variables along potential risk factors to be assessed during the donor evaluation.

The standardized psychometric instruments for the psychosocial assessment were chosen if a validated version in every participant center’s language was available, if norms for the general population had been defined, and on the basis of their reputation and acceptance in the clinical and scientific society (supplementary table 1). The following domains and instruments were included in the study:

- *Quality of life (QoL)* Anamnestic Comparative Self-Assessment (ACSA); 36-Item Short Form Survey (SF-36).
- *Mental health status* Hospital Anxiety Depression Scale (HADS).
- *Coping strategies* Dispositional Optimism (LOTR); Sense of Coherence scale (SOCS).
- *Personality* Eysenck Personality Questionnaire-Revised-Abbreviated (EPQ-RA).
- *Socio-economic status* English Longitudinal Study of Ageing (ELSA) Self-anchoring scale.

Considering that generic instruments might be unable to capture some of the subtleties of LKDT, specific ad hoc items regarding the donation process were extracted from previous studies^47–49^, and/or were designed for each outcome area (e.g. motivation for donation, risk assessment, decision-making process) (supplementary annex 1). The methodology to translate the ad hoc items, was similar for all the centers. First, one independent translation from English to each participant language were obtained from professional translators, who were native English speakers and bilingual in the center language. Second, the research team of each participant center and the translator agreed on a version conceptually equivalent to the original one.

The recipients’ clinical profile and outcome was examined in order to assess its potential influence on LKDs psychological outcomes. We collected data on pre-transplantation clinical parameters (e.g. African ancestry, primary chronic kidney disease), risks factors (e.g. high immunological risk, comorbid diseases), and recipient/graft 1-year complications (e.g. permanent kidney damage) (supplementary annex 2).

### Procedure

To ensure a proper implementation of the methodology, each participant center applied the same questionnaires and adapted the methodology to their characteristics and own resources (e.g. the creation of multidisciplinary teams inside the hospitals with the involvement of a psychologist). LKDs questionnaires were self-administrated.

#### Pre-donation

Approximately 1-month before donation, potential LKDs were informed face-to-face about the study by the responsible investigator. At this stage, they signed the informed consent form and received a printed questionnaire to be completed at home before the psychological evaluation.

#### Post-donation

Approximately 12-months after donation, LKDs were contacted by phone in advance and asked whether they would agree to receive the post-operative questionnaire via postal-mail. They were asked to return the questionnaire in a pre-paid envelope. LKDs that did not send the questionnaire back within a month were contacted again by phone.

The nephrologist responsible for the recipients’ care collected data regarding their clinical variables on standardized form (supplementary annex 2).

### Data management

An online database was developed to introduce the data about the study. The participant centers were responsible to introduce in the database their own data provided from each of the questionnaires. All the items were coding to facilitate the subsequent importation and analysis of the data.

### Statistical procedures

The factor structure of motivation for donation was assessed by principal component analysis, retaining those factors above the point where the slope goes from steep to flat in the scree-plot (elbow point)^50^. The internal consistency of the items forming each derived factor was assessed by Cronbach’s alphas.

LKDs that completed follow-up and those lost during the follow-up were compared to ensure that the cause of attrition was not related to LKDs’ characteristics or to the characteristics of their recipient.

First assessment: means at pre and post-donation of quantitative variables (e.g. SF-36, and HADS) were compared by paired t-test. Categorical variables were compared by the McNemar test.

Second assessment: the potential grouping of LKDs according to their level of HRQoL was assessed with a two-step cluster-analysis using the percentage of change observed in the SF-36 T-scores. Against the traditional k-means and hierarchical clustering approaches, two-step cluster allows using variables with different measurement levels or continuous with skewed distribution, and automatically determines the optimum number of clusters^51^. Mean or proportion differences between clusters were analyzed to characterize those donors with a HRQoL decrement.

This clustering of LKDs’ outcomes allowed for the selection of potential predictive variables of HRQoL decrement, which was done in two-steps: firstly, univariate logistic regression models with the group obtained in cluster-analysis as the dependent variable, and each of the potential risk factors as independent terms were estimated. The false discovery rate was applied to know what predictors kept statistical significance after correction^52^. Secondly, each predictor reaching a non-corrected statistical significance below 0.30 was selected as potential predictor for the next analysis^53^. A logistic regression model with forward stepwise selection based on the significance value was applied to obtain the final best predictive model of the clusters previously derived. The predictive capability of the final model was assessed through the percentage of total correct classifications, sensibility, specificity, and the area under the ROC curve (AUC). Because the limited sample size did not allow to validate results by splitting the sample, an internal validation with bootstrapping calculation was done to obtain AUC^54^.

#### Addendum

Previous publications have shown that the classical N/p rule-of-thumb does not perform very well in practice^55^. A simulation paper has addressed requirements of exploratory factor analysis with small sample sizes (even lower than N = 50)^56^, indicating that other consideration as the factor’s correlations, the quantity of secondary loadings, the gap size between eigenvalues or the communalities are more important to warranty the stability of the estimations. In our data, a 19% of the items load in more than one factor (none in 3 or more factors). The rotated eigenvalues are close to each other as revealed by the explained percentage of variance (15.6%, 14.2%, 12.4% and 11.5%), the communalities are quite homogeneous (range 0.23–0.77), and when estimating a non-orthogonal rotation allowing factors to correlate between them, the obtained correlations are low.

## Supplementary information

Supplementary Information 1.

Supplementary Information 2.

Supplementary Information 3.

## Data Availability

The data that support the findings of this study are available on request from the corresponding author.
