# Supplementary material for: Psychosocial risk factors for impaired health-related quality of life in living kidney donors: results from the ELIPSY prospective study
Source: Sci Rep. 2020 Dec 7;10:21343. doi: 10.1038/s41598-020-78032-8 (PMC7721886; doi:10.1038/s41598-020-78032-8)
Supplement: Supplementary file 1 — Supplementary Information 1. [file 41598_2020_78032_MOESM1_ESM.doc]

**• Title page**

**Title:**

Psychosocial risk factors for impaired health-related quality of life in living kidney donors – results from the ELIPSY prospective study

**Authors’ names:**

Ana Menjivar1,2^, Xavier Torres3^, Marti Manyalich^1,4^, Ingela Fehrman-Ekholm^5^, Christina Papachristou^6^, Erika de Sousa-Amorim^7^, David Paredes^2,8^, Christian Hiesse^9^, Levent Yucetin^10^, Federico Oppenheimer^2,7^, Entela Kondi^1,4^, Josep Maria Peri^3^, Niclas Kvarnström^11^, Chloë Ballesté^1^, Leonidio Dias^12^, Inês C Frade^13^, Alice Lopes^13^, Fritz Diekmann^2,7^, Ignacio Revuelta^1,2,7*^.

^ Both authors contributed in the same proportion.

**Authors’ institutional affiliations:**

1 Medical School, University of Barcelona, Barcelona, Spain

2 Laboratori Experimental de Nefrologia i Trasplantament (LENIT), Institut d’Investigacions Biomediques August Pi i Sunyer (IDIBAPS), Barcelona, Spain

3 Psychiatry and Clinical Psychology Service, Institut Clinic de Neurociencies, Hospital Clinic of Barcelona, Barcelona, Spain

4 Transplant Assessorial Unit, Medical Direction, Hospital Clinic of Barcelona, Barcelona, Spain

5 Karolinska Institutet, Transplantation Surgery, Karolinska University Hospital, Stockholm, Sweden

6 Department for Internal Medicine and Psychosomatics, Charité, University Medicine, Berlin, Germany

7 Department of Nephrology and Renal Transplantation, Hospital Clinic of Barcelona, Barcelona, Spain

8 Donation and Transplant Coordination Section, Hospital Clinic of Barcelona, Barcelona, Spain

9 Service de Néphrologie et de Transplantation Rénale, Hôpital Foch, Suresnes Cedex, France

10 Organ Transplant Coordination, Antalya Medical Park Hospital, Antalya, Turkey

11 Department of Transplantation, Institute of Clinical Sciences, Sahlgrenska Academy, University of Gothenburg, Sahlgrenska University Hospital, Gothenburg, Sweden.

12 Nephrology and Transplant Departments, Hospital Geral de Santo António, Porto, Portugal

13 Liaison-Psychiatry and Health Psychology Unit, Hospital Geral de Santo António, Porto, Potugal

**Contact information and corresponding author:**

Ignacio Revuelta

Hospital Clinic of Barcelona

Address: 170 Villarroel St. 12/5, Barcelona, Spain 08036

Phone: +34 639139850

Email: irevuelt@clinic.cat

**Supplementary annex 1**

**Specific ad hoc items regarding the donation process used**

**PREDONATION:**

**PSYCHIATRIC HISTORY**

Have you ever seen a counsellor, psychiatrist or psychologist? Yes 🔾 No 🔾

**MOTIVATION/REASONS FOR DONATION**

How important are the following considerations to you for taking the decision to donate your kidney?

|  | Not relevant | | |  | Very important | | |
| --- | --- | --- | --- | --- | --- | --- | --- |
| Wish to improve the recipient’s quality of life. | | 🔾 | 🔾 | 🔾 | | 🔾 | 🔾 |
| Fear that giving up my kidney might shorten my life span. | | 🔾 | 🔾 | 🔾 | | 🔾 | 🔾 |
| Wish to save the life of the recipient. | | 🔾 | 🔾 | 🔾 | | 🔾 | 🔾 |
| Fear that giving up my kidney might damage my health or cause me problems in the future. | | 🔾 | 🔾 | 🔾 | | 🔾 | 🔾 |
| Concern that some of my family or friends disapprove of my donating my kidney. | | 🔾 | 🔾 | 🔾 | | 🔾 | 🔾 |
| Concern that some of my family or friends would disapprove of my decision to not donate. | | 🔾 | 🔾 | 🔾 | | 🔾 | 🔾 |
| Wish to feel closer to the person that I was donating my kidney. | | 🔾 | 🔾 | 🔾 | | 🔾 | 🔾 |
| The belief that my recipient’s kidney disease was at least partly their fault as the result of bad health habits. | | 🔾 | 🔾 | 🔾 | | 🔾 | 🔾 |
| Concern about having a scar. | | 🔾 | 🔾 | 🔾 | | 🔾 | 🔾 |
| Concern about financial implications of donating my kidney. | | 🔾 | 🔾 | 🔾 | | 🔾 | 🔾 |
| Feeling that this was a unique opportunity to do something very special. | | 🔾 | 🔾 | 🔾 | | 🔾 | 🔾 |
| Wish to feel that I am a good person | | 🔾 | 🔾 | 🔾 | | 🔾 | 🔾 |
| Worry that the person I was donating my kidney might not take care of it properly. | | 🔾 | 🔾 | 🔾 | | 🔾 | 🔾 |
| I didn’t want to lose the recipient, I am afraid he/she will die if I don’t donate | | 🔾 | 🔾 | 🔾 | | 🔾 | 🔾 |
| Wish to have a nice life together with the recipient | | 🔾 | 🔾 | 🔾 | | 🔾 | 🔾 |
| I am unable to watch the recipient suffer and I don’t do anything about it | | 🔾 | 🔾 | 🔾 | | 🔾 | 🔾 |
| It is a personal challenge/test for me | | 🔾 | 🔾 | 🔾 | | 🔾 | 🔾 |
| Wish to show my deep love and respect to the recipient | | 🔾 | 🔾 | 🔾 | | 🔾 | 🔾 |
| Feeling responsible for the recipient | | 🔾 | 🔾 | 🔾 | | 🔾 | 🔾 |
| Feeling I owe that to the recipient | | 🔾 | 🔾 | 🔾 | | 🔾 | 🔾 |
| Wish not to disappoint the recipient | | 🔾 | 🔾 | 🔾 | | 🔾 | 🔾 |
| I want to protect other family members from pain and improve their family life | | 🔾 | 🔾 | 🔾 | | 🔾 | 🔾 |
| In past situations, I did not have the chance to influence things. Now I do and I want to use this chance | | 🔾 | 🔾 | 🔾 | | 🔾 | 🔾 |
| The recipient is too young to suffer/die and deserves to live further | | 🔾 | 🔾 | 🔾 | | 🔾 | 🔾 |
| Feeling obligated as a person/relative to do that | | 🔾 | 🔾 | 🔾 | | 🔾 | 🔾 |
| Feeling of being responsible as a family member to do that | | 🔾 | 🔾 | 🔾 | | 🔾 | 🔾 |
| I am the only donor available | | 🔾 | 🔾 | 🔾 | | 🔾 | 🔾 |
| I am the most suitable donor | | 🔾 | 🔾 | 🔾 | | 🔾 | 🔾 |
| There is no other choice | | 🔾 | 🔾 | 🔾 | | 🔾 | 🔾 |
| The family thought/decided that I would be the most suitable to donate | | 🔾 | 🔾 | 🔾 | | 🔾 | 🔾 |
| It just makes sense to me to donate | | 🔾 | 🔾 | 🔾 | | 🔾 | 🔾 |

**DECISION MAKING**

**Which of the following is true for you? Please check.**

When I heard about the possibility/need of the donation, I thought I need some time to think over it and get informed first

Strongly agree/agree 🔾 Disagree/strongly disagree 🔾

**RISK ASSESSMENT**

Do you feel you need more information regarding the surgery and the risks?

Yes 🔾 No 🔾

**POST-DONATION:**

**DONATION SPECIFIC QUESTIONS REGARDING THE QUALITY OF LIFE.**

Do you still suffer of any complaints or illnesses because of the donation (e.g. scar pains etc.)?

Yes 🔾 No 🔾

If yes, please specify ________________________________________________________

On a scale from zero to 10 how much do you think your current physical condition is affected by the donation? Please check.

| 0 | 1 | 2 | 3 | 4 | 5 | 6 | 7 | 8 | 9 | 10 |
| --- | --- | --- | --- | --- | --- | --- | --- | --- | --- | --- |

The postoperative recovery was

More difficult than I had imagined 🔾

Easier than I had imagined 🔾

Exactly as I had imagined 🔾

I do not know/I do not remember 🔾

**DONATION SPECIFIC QUESTIONS REGARDING MENTAL HEALTH**

Did you need psychological/psychiatric treatment or counselling since the donation?

Yes 🔾

Yes, because of the donation 🔾

No 🔾

On a scale from zero to 10 how much do you think your current emotional condition is affected by the donation? Please check.

| 0 | 1 | 2 | 3 | 4 | 5 | 6 | 7 | 8 | 9 | 10 |
| --- | --- | --- | --- | --- | --- | --- | --- | --- | --- | --- |

Please check whether the following statements apply to you or not?

|  | True | False |
| --- | --- | --- |
| After surgery I felt forgotten | 🔾 | 🔾 |
| Somehow I feel worried about my health since the donation | 🔾 | 🔾 |
| The recipients‘ health still occupies me a lot (please leave unchecked  if the recipient passed away) | 🔾 | 🔾 |

**SOCIO-ECONOMIC STATUS**

Are you employed at the moment?

Fully employed 🔾

Part-time 🔾

Part-time because of the donation 🔾

Unemployed 🔾

Unemployed because of the donation 🔾

Retired 🔾

Retired because of the donation 🔾

Homemaker 🔾

Student 🔾

Other _______________________ 🔾

Did you suffer any financial loss due to the donation?

Yes 🔾 No 🔾

**LIFE EVENTS**

Did you experience any other life changing events since donation, as marriage, death of a loved one, accident, illness, loss of job, divorce, exams, end of studies etc.?

Yes 🔾 No 🔾

Could you please specify them? Please, also rate at the right column the intensity of the distress caused to you by each of these events (please include the donation process)

| Event | Intensity of distress (0=no distress at all;  10=the worst experience in my life) | | | | | | | | | | |
| --- | --- | --- | --- | --- | --- | --- | --- | --- | --- | --- | --- |
| a. | 0 | 1 | 2 | 3 | 4 | 5 | 6 | 7 | 8 | 9 | 10 |
| b. | 0 | 1 | 2 | 3 | 4 | 5 | 6 | 7 | 8 | 9 | 10 |
| c. | 0 | 1 | 2 | 3 | 4 | 5 | 6 | 7 | 8 | 9 | 10 |
| d. | 0 | 1 | 2 | 3 | 4 | 5 | 6 | 7 | 8 | 9 | 10 |
| e. | 0 | 1 | 2 | 3 | 4 | 5 | 6 | 7 | 8 | 9 | 10 |
| f. | 0 | 1 | 2 | 3 | 4 | 5 | 6 | 7 | 8 | 9 | 10 |
| g. Donation process | 0 | 1 | 2 | 3 | 4 | 5 | 6 | 7 | 8 | 9 | 10 |

**DONOR-RECIPIENT RELATIONSHIP**

Do you feel your relationship to the recipient has changed after the donation?

Yes 🔾 No 🔾

Have you been in charge of the recipient since the donation?

Yes 🔾 No 🔾

**PERCEPTION OF THE RECIPIENT ‘S HEALTH AFTER TRANSPLANTATION**

The recipient enjoys good health currently

Strongly agree/agree 🔾 Disagree/strongly disagree 🔾

The recipient of my organ behaves in a way that risks the continued healthy functioning of the donated kidney

Strongly agree/agree 🔾 Disagree/strongly disagree 🔾
