# Supplementary material for: Psychosocial risk factors for impaired health-related quality of life in living kidney donors: results from the ELIPSY prospective study
Source: Sci Rep. 2020 Dec 7;10:21343. doi: 10.1038/s41598-020-78032-8 (PMC7721886; doi:10.1038/s41598-020-78032-8)
Supplement: Supplementary file 2 — Supplementary Information 2. [file 41598_2020_78032_MOESM2_ESM.doc]

**• Title page**

**Title:**

Psychosocial risk factors for impaired health-related quality of life in living kidney donors – results from the ELIPSY prospective study

**Authors’ names:**

Ana Menjivar1,2^, Xavier Torres3^, Marti Manyalich^1,4^, Ingela Fehrman-Ekholm^5^, Christina Papachristou^6^, Erika de Sousa-Amorim^7^, David Paredes^2,8^, Christian Hiesse^9^, Levent Yucetin^10^, Federico Oppenheimer^2,7^, Entela Kondi^1,4^, Josep Maria Peri^3^, Niclas Kvarnström^11^, Chloë Ballesté^1^, Leonidio Dias^12^, Inês C Frade^13^, Alice Lopes^13^, Fritz Diekmann^2,7^, Ignacio Revuelta^1,2,7*^.

^ Both authors contributed in the same proportion.

**Authors’ institutional affiliations:**

1 Medical School, University of Barcelona, Barcelona, Spain

2 Laboratori Experimental de Nefrologia i Trasplantament (LENIT), Institut d’Investigacions Biomediques August Pi i Sunyer (IDIBAPS), Barcelona, Spain

3 Psychiatry and Clinical Psychology Service, Institut Clinic de Neurociencies, Hospital Clinic of Barcelona, Barcelona, Spain

4 Transplant Assessorial Unit, Medical Direction, Hospital Clinic of Barcelona, Barcelona, Spain

5 Karolinska Institutet, Transplantation Surgery, Karolinska University Hospital, Stockholm, Sweden

6 Department for Internal Medicine and Psychosomatics, Charité, University Medicine, Berlin, Germany

7 Department of Nephrology and Renal Transplantation, Hospital Clinic of Barcelona, Barcelona, Spain

8 Donation and Transplant Coordination Section, Hospital Clinic of Barcelona, Barcelona, Spain

9 Service de Néphrologie et de Transplantation Rénale, Hôpital Foch, Suresnes Cedex, France

10 Organ Transplant Coordination, Antalya Medical Park Hospital, Antalya, Turkey

11 Department of Transplantation, Institute of Clinical Sciences, Sahlgrenska Academy, University of Gothenburg, Sahlgrenska University Hospital, Gothenburg, Sweden.

12 Nephrology and Transplant Departments, Hospital Geral de Santo António, Porto, Portugal

13 Liaison-Psychiatry and Health Psychology Unit, Hospital Geral de Santo António, Porto, Potugal

**Contact information and corresponding author:**

Ignacio Revuelta

Hospital Clinic of Barcelona

Address: 170 Villarroel St. 12/5, Barcelona, Spain 08036

Phone: +34 639139850

Email: irevuelt@clinic.cat

**Supplementary annex 2**

**1. CLINICAL EVALUATION OF THE RECIPIENT (BASELINE):**

Weight: ___________ (Kg)

African ancestry: Yes 🔾 No 🔾

Indication of transplantation (Primary chronic kidney disease):

______________________________________________________________________

Registration in waiting list for deceased donor: Yes 🔾 No 🔾

Dialysis before transplantation: Yes 🔾 No 🔾

Previous kidney transplant: Yes 🔾 No 🔾

Previous other organ transplant: Yes 🔾 No 🔾

High immunological risk: Yes 🔾 No 🔾

e.g. XM (+) Late PRA > 80% ABO incompatible Late graft lost of immune origin

High risk of recurrence: Yes 🔾 No 🔾

10- Presence of serious co-morbid diseases: Yes 🔾 No 🔾

*e.g. Previous cancer, previous cardiovascular event: event or medical intervention e.g. By-pass, stent*

**2.** **RECIPIENTS’ POST-TRANSPLANT COMPLICATIONS**

Permanent damage in organ function**:** Yes 🔾 No 🔾

Requires inpatient hospitalization or prolongation of existing hospitalization: Yes 🔾 No 🔾

Permanent or temporal discapacity not requiring hospitalization: Yes 🔾 No 🔾

Non–adherence: Yes 🔾 No 🔾

Deviation from the prescribed medication regimen sufficient to influence adversely the regimen’s intended effect

Psychological complications requiring treatment: Yes 🔾 No 🔾

Serum creatinine: __________ Units

Recipient surviving: Yes 🔾 No 🔾

Graft surviving: Yes 🔾 No 🔾
