# Supplementary material for: Psychosocial risk factors for impaired health-related quality of life in living kidney donors: results from the ELIPSY prospective study
Source: Sci Rep. 2020 Dec 7;10:21343. doi: 10.1038/s41598-020-78032-8 (PMC7721886; doi:10.1038/s41598-020-78032-8)
Supplement: Supplementary file 3 — Supplementary Information 3. [file 41598_2020_78032_MOESM3_ESM.docx]

## • Title page

## Title:

## Psychosocial risk factors for impaired health-related quality of life in living kidney donors – results from the ELIPSY prospective study

## Authors’ names:

Ana Menjivar^1,2^^, Xavier Torres^3^^, Marti Manyalich^1,4^, Ingela Fehrman-Ekholm^5^, Christina Papachristou^6^, Erika de Sousa-Amorim^7^, David Paredes^2,8^, Christian Hiesse^9^, Levent Yucetin^10^, Federico Oppenheimer^2,7^, Entela Kondi^1,4^, Josep Maria Peri^3^, Niclas Kvarnström^11^, Chloë Ballesté^1^, Leonidio Dias^12^, Inês C Frade^13^, Alice Lopes^13^, Fritz Diekmann^2,7^, Ignacio Revuelta^1,2,7*^.

^ Both authors contributed in the same proportion.

## Authors’ institutional affiliations:

1 Medical School, University of Barcelona, Barcelona, Spain

2 Laboratori Experimental de Nefrologia i Trasplantament (LENIT), Institut d’Investigacions Biomediques August Pi i Sunyer (IDIBAPS), Barcelona, Spain

3 Psychiatry and Clinical Psychology Service, Institut Clinic de Neurociencies, Hospital Clinic of Barcelona, Barcelona, Spain

4 Transplant Assessorial Unit, Medical Direction, Hospital Clinic of Barcelona, Barcelona, Spain

5 Karolinska Institutet, Transplantation Surgery, Karolinska University Hospital, Stockholm, Sweden

6 Department for Internal Medicine and Psychosomatics, Charité, University Medicine, Berlin, Germany

7 Department of Nephrology and Renal Transplantation, Hospital Clinic of Barcelona, Barcelona, Spain

8 Donation and Transplant Coordination Section, Hospital Clinic of Barcelona, Barcelona, Spain

9 Service de Néphrologie et de Transplantation Rénale, Hôpital Foch, Suresnes Cedex, France

10 Organ Transplant Coordination, Antalya Medical Park Hospital, Antalya, Turkey

11 Department of Transplantation, Institute of Clinical Sciences, Sahlgrenska Academy, University of Gothenburg, Sahlgrenska University Hospital, Gothenburg, Sweden.

12 Nephrology and Transplant Departments, Hospital Geral de Santo António, Porto, Portugal

13 Liaison-Psychiatry and Health Psychology Unit, Hospital Geral de Santo António, Porto, Potugal

**Contact information and corresponding author:**

Ignacio Revuelta

Hospital Clinic of Barcelona

Address: 170 Villarroel St. 12/5, Barcelona, Spain 08036

Phone: +34 639139850

Email: irevuelt@clinic.cat

**Supplementary table 1**

**DESCRIPTION OF THE STANDARDIZED PSYCHOMETRIC INSTRUMENTS FOR THE PSYCHOSOCIAL ASSESSMENT**

| **Quality of life:** | | |
| --- | --- | --- |
| Anamnestic Comparative Self-Assessment (ACSA) | Affected parameters and “state” risk factors | The ACSA is a self-anchoring rating scale for subjective well-being. It is a one-item of generic measure of quality of life. It has been recommended specially for longitudinal or intervention studies and for intercultural comparisons. The ACSA has shown to reduce biases due to casualness of responses and of proximate, peer or cultural bias and relativity by allowing people to define the endpoints or 'anchors' of the measurement scale. |
| 36-Item Short Form Survey (SF-36, RAND corporation, Santa Monica, CA) | Affected parameters and “state” risk factors | The SF-36 is a valid measure of health-related quality of life (HRQoL), in general and specific populations, comparing the relative burden of diseases, and in differentiating the health benefits produced by a wide range of different treatments. The SF-36 consists of eight scaled scores, which are the weighted sums of the questions in their section. Each scale is directly transformed into a 0-100 scale on the assumption that each question carries equal weight. The lower the score the more disability. The higher the score the less disability. |
| **Mental health status:** | | |
| Hospital Anxiety Depression Scale (HADS) | Considered both outcome variables and potential risk factors | The HADS assesses symptoms of depression and anxiety. Unlike other questionnaires designed to measure psychopathology, the HADS does not evaluate symptoms that may be due to a physical cause and, therefore, it is considered particularly suitable for people with medical illnesses. HADS can be used to screen outpatients for potential anxiety and depressive disorders. The HADS questionnaire has seven items each for depression and anxiety subscales. Scoring for each item ranges from zero to three, with three denoting highest anxiety or depression level. We expected that higher anxiety and depression punctuations among LKDs with a post-donation reduction of their HRQoL. |
| **Coping strategies**: | | |
| Dispositional Optimism (LOT-R) | Potential risk factor | The LOT-R measures optimism, which is a strong predictor of effective coping in stressful circumstances and identifies individuals who maintain positive expectations on adversity. The LOT-R is a 10-item scale that measures how optimistic or pessimistic people feel about the future. Scoring for each item ranges from zero to four (“strongly disagree” to “strongly agree”), four items are “filler” statements that are not scored. Higher scores indicating optimism. Higher optimism and lower mental health have been associated with expected benefits of donation, whereas expected consequences of donation were associated with lower optimism and lower mental health^^[[1]](#footnote-1)^^. We expected that lower dispositional optimism punctuations among LKDs with a post-donation reduction of their HRQoL. |
| Sense of Coherence scale (SOCS) | Potential risk factor | The SOCS is a scale that assesses how people view the life and a scale that seeks to identify how people might use their resources to overcome resistance and to maintain and develop their health. A concept developed and established by Antonovsky, as a central construct of his theory of salutogenesis. A shorter version of 13-items were used with the score ranges between 13 and 91 points. The higher the total score of the SOC, the stronger is the sense of coherence. The SOCS has proven to be a significant predictor for anxiety, depression and mental quality of life of donors, prior transplantation^^[[2]](#footnote-2)^^. We expected lower SOCS scores in LKDS with lower post-donation HRQoL scores. |
| **Personality:** | | |
| Eysenck Personality Questionnaire-Revised-Abbreviated (EPQ-RA) | Potential risk factor | Associations have been reported between personality traits and several health behaviors (behaviors that reduce the risk of overtaxing the body’s adaptive capacity; behaviors that involve reducing risk-taking; and behaviors that could improve health rather than merely prevent illness and health outcomes). The EPQ-RA is a 24-items self-administered questionnaire assesses four scales of six items each (extraversion, neuroticism, psychoticism, and the lie scale). We expected significant positive correlations between neuroticism and lower HRQoL, and non-significant correlations between the other personality dimensions and the HRQoL. |
| **Socio-economic status:** | | |
| English Longitudinal Study of Ageing (ELSA) Self-anchoring scale | Considered both outcome variables and potential risk factors | An individual’s subjective assessment of their social status is a powerful predictor of their health status regardless the gender. Data suggest that there is a higher prevalence of angina, diabetes, poor self-rated-health, and depression amongst individuals who rate themselves as having low social status as compared to those who see themselves as having higher status. In LKDS, the availability of, for instance, financial resources that could cover unexpected costs in comparison to limited financial capacity to manage donation (lost wages, travel) has been shown as a protective factor while a reduction in the self-perceived social status (due for instance to an unaffordable economic burden due to donation) might be considered an adverse psychosocial outcome. We expected that LKDS with a post-donation reduction of their socio-economic status will be those with affectation of their HRQoL. |

1. Rodrigue JR, Guenther R, Kaplan B, Mandelbrot DA, Pavlakis M, Howard RJ. Measuring the expectations of kidney donors: initial psychometric properties of the Living Donation Expectancies Questionnaire. Transplantation. 15, 1230-4, http://doi.org/10.1097/TP.0b013e31816c5ab0 (2008) [↑](#footnote-ref-1)
2. Erim Y, et al. Sense of coherence and social support predict living liver donors' emotional stress prior to living-donor liver transplantation. Clin Transplant. 22, 273-80, http://doi.org/10.1111/j.1399-0012.2007.00782.x (2008). [↑](#footnote-ref-2)
